# Gene set analysis using variance component tests

**DOI:** 10.1186/1471-2105-14-210

**Published:** 2013-06-28

**Authors:** Yen-Tsung Huang, Xihong Lin

**Affiliations:** 1 Department of Epidemiology, Brown University, 121 South Main Street, Providence, RI 02912, USA; 2 Department of Biostatistics, Harvard School of Public Health, 655 Huntington Ave, Boston, MA 02115, USA

## Abstract

**Background:**

Gene set analyses have become increasingly important in genomic research, as many complex diseases are contributed jointly by alterations of numerous genes. Genes often coordinate together as a functional repertoire, e.g., a biological pathway/network and are highly correlated. However, most of the existing gene set analysis methods do not fully account for the correlation among the genes. Here we propose to tackle this important feature of a gene set to improve statistical power in gene set analyses.

**Results:**

We propose to model the effects of an independent variable, e.g., exposure/biological status (yes/no), on multiple gene expression values in a gene set using a multivariate linear regression model, where the correlation among the genes is explicitly modeled using a working covariance matrix. We develop TEGS (Test for the Effect of a Gene Set), a variance component test for the gene set effects by assuming a common distribution for regression coefficients in multivariate linear regression models, and calculate the p-values using permutation and a scaled chi-square approximation. We show using simulations that type I error is protected under different choices of working covariance matrices and power is improved as the working covariance approaches the true covariance. The global test is a special case of TEGS when correlation among genes in a gene set is ignored. Using both simulation data and a published diabetes dataset, we show that our test outperforms the commonly used approaches, the global test and gene set enrichment analysis (GSEA).

**Conclusion:**

We develop a gene set analyses method (TEGS) under the multivariate regression framework, which directly models the interdependence of the expression values in a gene set using a working covariance. TEGS outperforms two widely used methods, GSEA and global test in both simulation and a diabetes microarray data.

## Background

Genome-wide analysis using microarray data, including RNA expression, DNA copy number and epigenetic DNA methylation, has become a popular tool in genomic research. Single gene/marker analysis provides a quick and convenient tool to identify top genes that might be associated with phenotypic trait. However, it is subject to a large number of false positives due to a large number of comparisons, and does not fully take into account that some genes have similar biological functions and work together.

Microarray gene expressions or genetic markers usually have natural groupings based on biological knowledge. For example, multiple genes belong to the same biological pathway or network; or contiguous copy number-detecting probes belong to the same gene or cytoband. Incorporating the prior knowledge or annotation about the grouping underlying the genome-wide data can make the results more interpretable. Note that the grouping may not necessarily come from biology. It can also be a cluster of genes identified using clustering methods. In this paper, these natural or statistical groupings are loosely called *a gene set*, which refers to a set of genes, or a set of markers or simply a set of probes.

Numerous approaches for gene set analyses have been proposed [1], including the overrepresentation analysis [2], the univariate tests [3], the multivariate tests [4,5], the global test [6], and gene set enrichment analysis (GSEA) [7,8] and its variant [9]. The overrepresentation-type analysis has been found to suffer from methodological problems, which may lead to confusing results [10]. The global test and GSEA improve over the overrepresentation-type analysis. The global test regresses the phenotype on gene expressions in a gene set and tests for regression coefficients. GSEA performs a modified Kolmogorov-Smirnov test by comparing a gene set with the rest of the genes in the genome. However, the test statistics used in both methods ignore the correlation among the genes in a gene set and hence are subject to loss of statistical power, as genes in a gene set are often correlated and function together. The univariate test does not account for the correlation and loses power when the interdependence within the gene set is high, compared with the multivariate tests [11].

We propose in this paper to test for the effect of a gene set using a variance component test in multivariate regression model, where the correlation among genes in a gene set is explicitly taken into account. We term this test TEGS (Test for the Effect of a Gene Set). Specifically, we regress the gene expressions in a gene set on an independent variable, such an exposure or biological state variable, e.g., smoking (yes/no) or lung cancer status (yes/no), using multivariate regression, where the correlation among genes in a gene set is modeled using a working covariance matrix. As the number of genes might be large in a gene set, we develop a variance component score test for testing the effects of the exposure/biological state on the overall gene set profile by assuming regression coefficients follow a common distribution.

We show that TEGS includes the global test of Goeman, *et al* (2004) as a special case when correlation among the genes in a gene set is ignored. We conduct simulation studies to evaluate the finite sample performance of TEGS and compare it with the global test and GSEA. We apply the proposed method to analysis of the Type II Diabetes data set [7].

## Methods

### The model

Suppose that there are *n* subjects and subject *i* has *p* continuous outcomes *Y*_*i*1_,*Y*_*i*2_,…,*Y*_*ip*_. In gene set analysis, the *p* outcomes indicate the expression values of *p* genes in a gene set, and *x*_*i*_ is an independent variable, e.g., exposure/biological state variable, such as mutation status: 1 if mutant and 0 if wild-type; or disease status (yes/no) for subject *i*. We consider the multivariate linear model

(1) $$Y_{\text{ij}}=\alpha_{j}+x_{i}\beta_{j}+\varepsilon_{\text{ij}},\;i=1,2,\mathrm{...},n\;\text{and}\;j=1,2,\mathrm{...},p$$

where the errors, ***ε***_*i*_ = (*ε*_*i*1_,*ε*_*i*2_,...,*ε*_*ip*_)^*T*^ are assumed to be independent across different subjects and follow a multivariate normal distribution with mean **0** and true covariance **Σ**, which is often unknown, and *α*_*j*_ is the average expression value of gene *j* for those with *x* = 0. Covariates can be incorporated in the model (1) by expanding *α*_*j*_ to be $\overset{K}{\underset{k=1}{\sum }}\alpha_{\text{jk}}z_{\text{ik}}$ where *K* is the number of covariates plus one (i.e., the intercept), *z*_*ik*_ is the covariate *k* of subject *i*, *z*_*i*1_ is 1, and *α*_*jk*_ is the regression coefficient of the covariate *k* for the gene *j*. However, because the data we are dealing with has small *n* and large *p*, we would need the ridge regression to estimate *α*_*jk*_. If *x*_*i*_ is binary, e.g., disease status, *β*_*j*_ is the mean difference of the expression levels of gene *j* between the two disease groups. Model (1) can be written in matrix notation by stacking data of *n* subjects and *p* gene expressions as

(2) $$\begin{array}{c} Y=J\alpha +X\beta +\varepsilon , \end{array}$$

where $Y\;=\;{\left(Y_{1}^{T},\cdots \;,Y_{n}^{T}\right)}^{T}$ is an *np* × 1 vector, ***Y***_*i*_ = (*Y*_*i*1_, *Y*_*i*2_,…,*Y*_*ip*_)^*T*^, $\varepsilon \;=\;{\left(\varepsilon_{1}^{T},\;\cdots \;,\varepsilon_{n}^{T}\right)}^{T}$, ***J*** = (***I***_*p*_, ⋯,***I***_*p*_)^*T*^,***X*** = (*x*_1_***I***_*p*_, ⋯,*x*_*n*_***I***_*p*_)^*T*^, ***α*** = (*α*_1_,*α*_2_, ⋯,*α*_*p*_)^*T*^, ***β*** = (*β*_1_,*β*_2_, ⋯,*β*_*p*_)^*T*^.

### Gene set analysis using TEGS: A variance component score test

The null hypothesis *H*_0_ : ***β*** = 0 indicates that *x*_*i*_ has no effect on the mean of gene expression profile ***Y***_*i*_ in a gene set. A traditional multivariate test for *H*_0_[4] is based on a *p*-degree of freedom test and hence has limited power when the size of the gene set *p* is large, especially in the presence of a large number of null genes. To overcome this problem and improve test power, we assume the regression coefficients *β*_*j*_ follows an arbitrary common distribution with mean 0 and variance *τ*. The model (2) becomes a linear mixed model [12]. The null hypothesis *H*_0_ : ***β*** = **0** is equivalent to the null hypothesis for the variance component *H*_0_ : *τ* = 0. To test for *H*_0_ : *τ* = 0, one can perform a variance component score test [13].

Specifically, following Lin (1997), simple calculations show that the score for the variance component *τ* under the induced linear mixed model is

(3) $${\left(Y-J\alpha \right)}^{T}\Sigma_{n}^{-1}{\mathrm{XX}}^{T}\Sigma_{n}^{-1}(Y-J\alpha )-\text{tr}\left(\Sigma_{n}^{-1}{\mathrm{XX}}^{T}\right),$$

where **Σ**_*n*_ = *diag*(**Σ**,⋯,**Σ**) is an *np* × *np* block diagonal matrix. As the second term does not depend on data, we use the first term to construct the test statistic

(4) QT=(Y−Jα^)TΣn−1XXTΣn−1(Y−Jα^),

where α^ is the maximum likelihood estimator of ***α*** under *H*_0_. One can easily show that under *H*_0_, α^=(JTΣn−1J)−1JTΣn−1Y=Y¯, where $\overset{¯}{Y}=n^{-1}\overset{n}{\underset{i=1}{\sum }}Y_{i}$ is simply the sample mean. Hence Equation (4) can be written as

$$Q_{T}=Y^{T}(I-H)\Sigma_{n}^{-1}{\mathrm{XX}}^{T}\Sigma_{n}^{-1}(I-H)Y,$$

where ***H*** = *n*^−1^***JJ***^*T*^. As *Q*_*T*_ is quadratic in ***Y***. Some calculations show that *Q*_*T*_ follows a mixture of chi-square distribution $\underset{j}{\sum }\lambda_{j}\chi_{1,j}^{2}$, where the weights *λ*_*j*_ are the eigenvalues of the matrix $X^{T}(I-H)\Sigma_{n}^{-1}(I-H)X$.

The test statistic *Q*_*T*_ depends on the true covariance matrix **Σ** of ***Y***_*i*_, which is often unknown in practice and requires estimation of a large number of parameters. Although sample covariance can be used to estimate **Σ**, it is not stable when the size of the gene set *p* is large or moderate and sample size is small. We hence propose the use of a working covariance ***V*** for ***ε***_*i*_ in (1) [14], which has a simpler structure and depends on a small number of parameters. We derive a variance component test for *H*_0_ : *τ* = 0 assuming ***ε***_*i*_ has a covariance ***V***, which might misspecify the true covariance **Σ**. Under this working model, similar calculations show that the variance component score statistic for *H*_0_ : *τ* = 0 is

(5) $$Q=Y^{T}(I-H)V_{n}^{-1}{\mathrm{XX}}^{T}V_{n}^{-1}(I-H)Y,$$

where ***V***_*n*_ = *diag*(***V***,⋯,***V***). We term the variance component test using *Q* TEGS (Test for the Effect of a Gene Set).

Examples of working covariance ***V*** include working independence (Indpt), which assumes the genes are independent in a gene set; factor analysis covariances assuming two factors (F-2); adaptive factor analysis covariance with the estimated number of factors explaining up to 80% variability (F-adpt), compound symmetry (CpSym), which assumes the same pair-wise correlation among genes; and unstructured sample covariance (Unstr).

The unstructured sample covariance is estimated using the residuals ${\hat{\varepsilon }}_{\text{ij}}$ obtained by performing separate simple linear regression of individual gene expressions ***Y***_*ij*_ on *x*_*i*_ in (1). When *x*_*i*_ is binary, e.g., disease=yes/no, ${\hat{\varepsilon }}_{\text{ij}}$ is simply the *j*th centered outcome using the group specific means. When the number of genes in a gene set is large and the sample size is small, the standard empirical unstructured sample covariance estimator is unstable. We hence stabilize it using a ridge estimator by adding the 5th percentiles of sample variances to the diagonal of the empirical covariance estimator. Estimation for the compound symmetry covariance and the factor analysis covariance was based on the ridge unstructured covariance estimator. Specifically, for the compound symmetry covariance estimator, the pair-wise covariance is estimated as the sample mean of the off-diagonal elements of the ridge unstructured covariance estimator. The two-factor and adaptive-factor covariances are estimated by singular value decomposition of the ridge unstructured covariance estimator.

We discuss in Section Null distribution of TEGS estimation of the p-value using the TEGS test statistic *Q*. We perfromed simulation studies to investigate the performance of size and power using different working covariances in a wide range of scenarios and compare TEGS with that using *Q*_*T*_, which is based on the true covariance matrix of ***Y***_*i*_ and is the optimal test statistic within the TEGS statistic family, but cannot be calculated in practice as the true covariance of ***Y***_*i*_ is unknown.

### Special case of two group comparison and relationship of TEGS with the global test

Consider the setting of testing for the effect of a binary exposure/disease status on expressions in a gene set, i.e., *x*_*i*_ is binary (0/1), some calculations show that the TEGS statistic *Q* in (5) can be simplified as

(6) $$\begin{array}{ll} \;Q & ={\left\{\bar{Y}(1)-\bar{Y}(2)\right\}}^{T}V_{n}^{-1}V_{n}^{-1}\{\bar{Y}(1)-\bar{Y}(2)\}\\ & ={\left\{\frac{n_{1}n_{2}}{n}\right\}}^{2}\overset{p}{\underset{j=1}{\sum }}{\left\{\overset{p}{\underset{k=1}{\sum }}v^{\text{jk}}[{\bar{Y}}_{k}(1)-{\bar{Y}}_{k}(2)]\right\}}^{2}, \end{array}$$

where $\bar{Y}(1)$ and $\bar{Y}(2)$ are the sample mean of the outcome vector for group 1 and 2, and ${\bar{Y}}_{k}(1)$ and ${\bar{Y}}_{k}(2)$ (*k* = 1,⋯,*p*) are their components, *v*^*jk*^ is the (*j*,*k*)th element of ***V***^−1^. This suggests that the TEGS statistic *Q* compares the weighted average of the outcome-specific mean differences of the gene expression profiles between the two groups.

If one assumes working independence ***V*** = ***I***, the TEGS test statistic *Q* in (6) becomes

(7) $$\begin{array}{ll} \;Q_{\text{ind}} & ={\left\{\bar{Y}(1)-\bar{Y}(2)\right\}}^{T}\{\bar{Y}(1)-\bar{Y}(2)\}\\ & ={\left\{\frac{n_{1}n_{2}}{n}\right\}}^{2}\overset{p}{\underset{k=1}{\sum }}{\left\{{\bar{Y}}_{k}(1)-{\bar{Y}}_{k}(2)\right\}}^{2}. \end{array}$$

It can be easily shown that the TEGS statistic that uses the working independence covariance among gene expressions in a gene set (*Q*_*ind*_) is the same as the global test of Goeman, *et al* (2004). Although the global test is equivalent to the TEGS with working independence, it is still derived under the model (1) where the true covariance Σ is not necessarily independent.

Specifically, the global test is derived as the variance component test under the logistic regression model of the binary disease status *x*_*i*_ on the *p* gene expressions

(8) $$\text{logit}(\pi_{i})=\gamma_{0}+\gamma_{1}Y_{i1}+\cdots +\gamma_{p}Y_{\text{ip}}.$$

where *π*_*i*_ = *Pr*(*x*_*i*_ = 1|*Y*_*i*1_,⋯,*Y*_*ip*_) is the probability of disease given the gene expression profiles in a gene set. Under the logistic model (8), to test for the null hypothesis of no gene set effect on disease status *H*_0_ : *γ*_1_ = ⋯ = *γ*_*p*_ = 0, Goeman, *et al* (2004) assumed the coefficients *γ*_*j*_ are independent and follow an arbitrary distribution with mean 0 and variance *τ*. The logistic model (8) hence becomes a logistic mixed model [15]. It follows that the null hypothesis *H*_0_ : *γ*_1_ = ⋯ = *γ*_*p*_ = 0 is identical to *H*_0_ : *τ* = 0. Goeman, *et al* (2004) derived the variance component score test for *H*_0_ : *τ* = 0 and termed it as the global test. One can easily show that the global test is identical to *Q*_*ind*_ in (7), apart from a term that does not depend on ***Y***.

A comparison of (6) and (7) shows that TEGS has the flexibility to account for different correlations among gene expressions in a gene set by comparing the weighted differences of the means of gene expressions between two groups, while the global test, which is the same as the TEGS assuming working independence among gene expressions, ignores correlation among gene expressions. One hence would expect that TEGS that accounts for within gene set correlation is likely to be more powerful than the global test.

Another testing procedure that is closely related to TEGS is the Sequence Kernel Association Test (SKAT), a method developed to analyze SNP (single nucleotide polymorphism) or sequence data in genome-wide association studies [16]. It has been shown that the global test is equivalent to the SKAT with linear kernel [16,17]. Thus, the TEGS with working independence is equivalent to the SKAT with linear kernel. However, TEGS with other working correlations and SKAT with other kernels do not have an obvious correspondence.

### Null distribution of TEGS

As the TEGS statistic *Q* in (5) is a quadratic function of ***Y***, we have shown that it follows a mixture of chi-square distributions, where the weights depend on the true covariance **Σ** and the working covariance ***V***. We propose two methods to estimate the p-value of TEGS.

#### Permutation

One approach to calculate the p-value for the TEGS statistic *Q*, is based on permutation, where we permute the *x*_*i*_’s, and calculate *Q* for each permuted dataset and compare the observed value of *Q* with those calculated based on the permuted samples. Note that for each permutation, ***V*** need to be re-estimated given an assumed structure, e.g., under independence, unstructured, exchangeable, as ***V*** is the covariance conditional on the *x*. If the sample size is large (i.e. >100), one may use the Monte Carlo approach proposed by Lin [18].

#### Scaled ***χ***^***2***^ approximation

The second approach is to compute the p-value for the TEGS statistic *Q* is to use the Satterthwaite method [19] to approximate the null distribution of *Q*, which is a mixture of *χ*^2^ distributions. The Satterthwaite method approximates the null distribution of *Q* by a scaled *χ*^2^ distribution $\kappa \chi_{\nu }^{2}$, where *κ* is the scale parameter and *ν* is the degree of freedom. The values of *κ* and *ν* can be estimated by matching the first two moments of *Q* under *H*_0_ with those of the the scaled *χ*^2^ distribution as

$$\kappa =\frac{\text{Var}(Q)}{2E(Q)},\nu =\frac{2{\left[E(Q)\right]}^{2}}{\text{Var}(Q)}.$$

We estimate the mean and variance of *Q* under the null using permutation and denote the p-value estimated using this approach as $p_{\kappa \chi_{\nu }^{2}}$. Using the Satterthwaite approximation, we are able to calculate small p-values based on a smaller number of permutations than the first method.

#### Normal mixture approximation

In order to achieve better precision of smaller p-values, we further propose a method using the normal mixture approximation [20]. Specifically, we fit a two-population normal mixture *π*_1_*N*(*μ*_1_,*σ*12) + *π*_2_*N*(*μ*_2_,*σ*22) for the $\Phi^{-1}(p_{\kappa \chi_{\nu }^{2}}^{\left(b\right)})$ where $p_{\kappa \chi_{\nu }^{2}}^{\left(b\right)}$ is the scaled *χ*^2^ approximated p-value for the statistic *Q*^(*b*)^ obtained at permutation *b*, *b* = 1,...,*B* (*B* is the number of permutation), Φ is the cumulative distribution function of the standard normal, and *π*_*a*_, *μ*_*a*_ and $\sigma_{a}^{2}$ are proportion, mean and variance of the normal distribution *a* (*a* = 1,2), respectively. p-value can then be estimated as the tail probability by comparing $\Phi^{-1}(p_{\kappa \chi_{\nu }^{2}})$ and ${\hat{\pi }}_{1}N({\hat{\mu }}_{1},{\hat{\sigma }}_{1}^{2})+{\hat{\pi }}_{2}N({\hat{\mu }}_{2},{\hat{\sigma }}_{2}^{2})$ where ${\hat{\mu }}_{a}$${\hat{\sigma }}_{a}^{2}$ and ${\hat{\pi }}_{a}$, respectively are maximum likelihood estimates of *μ*_*a*_, $\sigma_{a}^{2}$ and *π*_*a*_.

### Power calculations

To design a new study using a gene set analysis, one needs to perform power calculations. We discuss in this section power calculations using TEGS. The distribution of *Q* under the alternative hypothesis follows a mixture of non-central chi-square distributions. We approximate this distribution using a scaled non-central chi-square distribution $\kappa \chi_{\nu }^{2}(\delta )$. Specifically, we first estimate *κ* and *ν* under *H*_0_ as $\kappa ={\text{Var}}_{H_{0}}(Q)/[\;2E_{H_{0}}(Q)]$ and ${\nu ={2[\;E}_{H_{0}}(Q)]}^{2}/{\text{Var}}_{H_{0}}(Q)$, where $E_{H_{0}}(Q)$ and ${\text{Var}}_{H_{0}}(Q)$ are the mean and the variance estimated theoretically as under the null as

$$\begin{array}{lll} \;E_{H_{0}}(Q) & =\text{tr}\left((I-H)V^{-1}{\mathrm{XX}}^{T}V^{-1}(I-H)\Sigma \right)\; & \;\\ \;{\text{Var}}_{H_{0}}(Q) & =2\text{tr}\left((I-H)V^{-1}{\mathrm{XX}}^{T}V^{-1}(I-H)\Sigma \right)\; & \;\\ & \;\left((I-H)V^{-1}{\mathrm{XX}}^{T}V^{-1}(I-H)\Sigma \right).\; & \; \end{array}$$

For power calculations, to estimate the non-centrality parameter *δ*, the theoretical mean $E_{H_{A}}(Q$) under the alternative is

$$\begin{array}{lll} \;E_{H_{A}}(Q) & =\text{tr}\left((I-H)V^{-1}{\mathrm{XX}}^{T}V^{-1}(I-H)\Sigma \right)\; & \;\\ & \;+\beta^{T}X^{T}(I-H)V^{-1}XX^{T}V^{-1}(I-H)X\beta .\; & \; \end{array}$$

Setting $E_{H_{A}}(Q)=(\nu +\delta )\kappa$, which is the mean of $\kappa \chi_{\nu }^{2}(\delta )$, one can solve for *δ*, and calculate the power of the test using $\text{Pr}(\chi_{\nu }^{2}(\delta )>\chi_{\nu ,\alpha }^{2})$ where *α* is the size of the test. The true covariance Σ and the working covariance ***V*** can be estimated using the pilot data. One can perform calculations by varying and the effects ***β***.

### Simulation study

#### Single gene set

We simulated the data using model (1). Two different combinations of *n* and *p* were considered: *n* = 50 and *p* = 10 and *n* = 20 and *p* = 40. Four different true covariances of ***ε***_*i*_, **Σ**, were investigated: (1) compound symmetry (CS), where we assumed the diagonal elements equal to 1 and the off-diagonal elements equal to 0.1 or 0.5; (2) first-order autoregressive (AR1), where we assumed the diagonal elements equal to 1 and off-diagonal elements decay by a factor of 0.5 or 0.8; (3) two factor covariance (F2): $\Sigma =P_{1}P_{1}^{T}+P_{2}P_{2}^{T}+\text{diag}\{u\}$, where the *p* elements of the two factors, **P**_1_ and **P**_2_ were generated independently from two Gaussian distributions, and **u** was chosen to make the diagonal elements of the **Σ** equal to 1’s; (4) the unstructured covariance (UNS), which was assumed to be the sample covariance of MAP00240_Pyrimidine_metabolism (*p* = 40) using the Type II Diabetes data in Mootha *et al.* (2003). The sample covariance of MAP00240_Pyrimidine_metabolism was calculated based on 17 subjects with normal glucose tolerance and 17 Type II Diabetes patients by conditioning on the disease status. To avoid singularity of the sample covariance, the 5th percentile of the diagonal elements was added to the diagonal to construct the true covariance matrix used in simulations.

**Figure 1:**
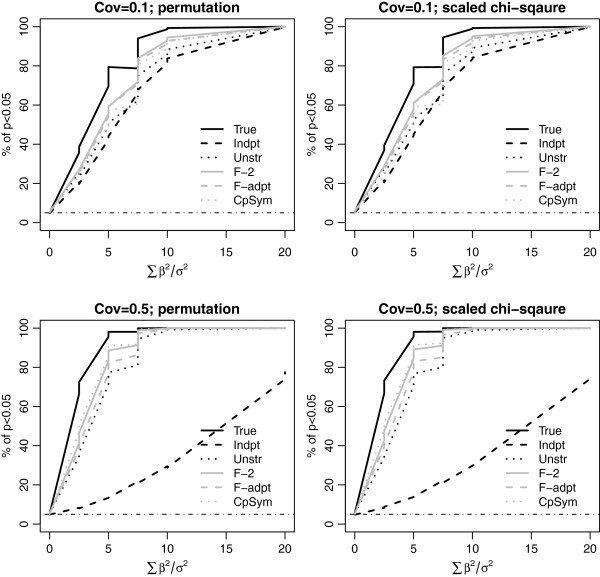
**Power comparison using simulations with the true covariance to be compound symmetry.** We set *n* = 20 and *p* = 40; covariance (cov) = 0.1 (top panel) and 0.5 (bottom panel). For each panel, the left plot is based on permutation and the right plot is based on the Satterthwaite scaled chi-square approximation. For each plot, the powers of TEGS assuming six different covariances were compared. TEGS assuming working independence (Indpt) corresponds to the global test of Goeman *et al.* (2004). The horizontal dotdash line indicates the size of the test (i.e., 5%).

**Figure 2:**
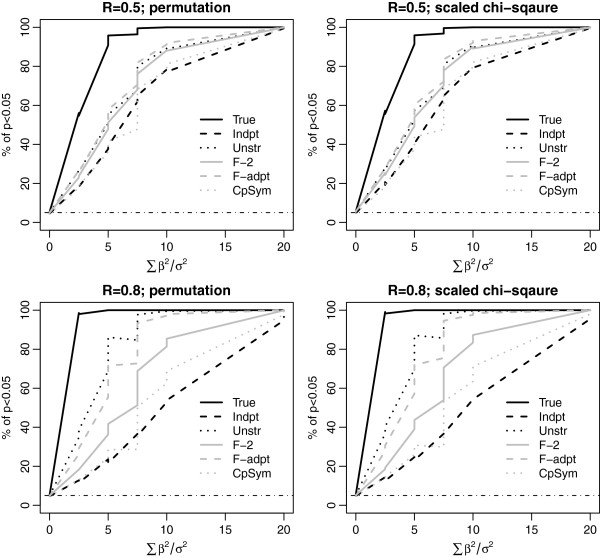
**Power comparison using simulations with the true covariance to be AR1.** R is the value of the lag 1 autocorrelation. We set *n* = 20 and *p* = 40; covariance (cov)=0.1 (top panel) and 0.5 (bottom panel). Other notations and symbols are similar to Figure 1.

**Figure 3:**
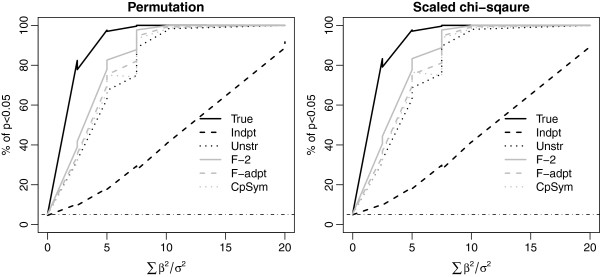
**Power comparison using simulations with the true covariance to be two factor covariance.** We set *n* = 20 and *p* = 40. Other notations and symbols are similar to Figure 1.

**Figure 4:**
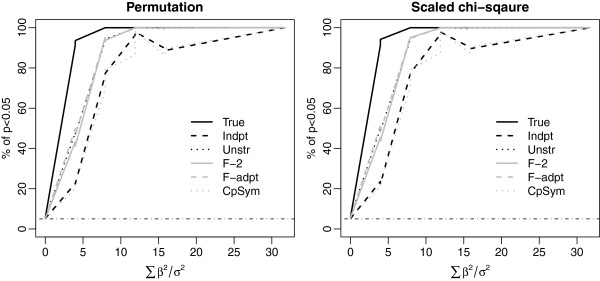
**Power comparison using simulations with the true covariance to be the stabilized sample covariance of MAP00240_Pyrimidine_metabolism.** We set *n* = 20 and *p* = 40. Other notations and symbols are similar to Figure 1.

The regression coefficients ***β*** was set by varying the proportion of non-zero *β*’s and their magnitudes. For *n* = 50 and *p* = 10, 0%, 40% and 80% of *β*’s were set to non-zero. The non-zero *β*s were set to be *β* = ±0.25 or ±0.5. For *n* = 20 and *p* = 40, 0%, 25%, 50% and 60% of *β*’s were set to be non-zero. The non-zero *β*s were set to be ±0.5 or ±1.0. The numbers of (−0.5,0.5) (or (−1.0,1.0)) are (2,8), (5,5), (5,15), (10,10), (5,25), (10,20) and (15,15). The effect size is summarized by an index, $\overset{p}{\underset{j=1}{\sum }}\beta_{j}/\bar{\sigma^{2}}$ where $\bar{\sigma^{2}}$ is the average variance of the *p* gene expression in the same gene set, in the power plots given in Figures 1, 2, 3 and 4.

For each simulation and each true covariance configuration, we compared the performance of TEGS assuming six different covariance matrices: true covariance, unstructured covariance, independence, two factor analysis covariance, adaptive factor analysis covariance, and compound symmetry. Note that the TEGS assuming working independence corresponds to the global test (Goeman *et al.* 2004). The p-values were calculated as the tail probability of *Q* under the null distribution. The null distribution was approximated by the methods described in Section Null distribution of TEGS. A total of 1000 permutations were performed to nonparametrically approximate the null distribution of *Q*. A total of 5000 and 1000 simulations, respectively were run for the setting under the null hypothesis (i.e., ***β*** = **0**) and the alternative hypothesis to calculate sizes and powers. Type I error was calculated at the *α* = 0.05 level. Statistical power was calculated as the percentage of p-values less than 0.05 among 1000 simulations.

#### Multiple gene sets

Gene set enrichment analysis (GSEA) is a widely used approach to study the enrichment of a gene set in a large number of genes, which often consists of multiple gene sets. The null hypothesis hence corresponds to the competitive null hypothesis [10]. To compare the performance of our proposed method TEGS with GSEA, we set up a simulation study involving multiple gene sets. The configuration is as follows: 

• *Setting 1*: We set *n* = 20 and the number of gene sets be 20. Ten gene sets have *p* = 10 genes (gene sets #1-10). Among them, six gene sets are under the null and four gene sets are under the alternative. The other ten gene sets have *p* = 40 genes per gene set (gene sets #11-20). Among them, six gene sets are under the null and four gene sets are under the alternative. Among the gene sets under the alternatives, we allowed some genes to have no effects (i.e., those with *β*_*j*_ = 0), and varied the number of signal genes (i.e., those with *β*_*j*_ ≠ 0). The number and magnitude of non-zero *β*’s or each of the gene sets under the alternative hypothesis are given in the top of Table 1. This setting has a total of 500 genes, with the total number of signal genes equal to 104 spreading across 8 gene sets and the total number of null genes equal to 396. We assumed in this setting the 20 gene sets were uncorrelated. Within each gene set, we assumed the genes were correlated with the two factor covariance matrix: $v^{*}=P_{1}P_{1}^{T}+P_{2}P_{2}^{T}+\text{diag}\{u\}$.

• *Setting 2*: This setting is identical to Setting 1 except that we allowed correlation among the gene sets: gene sets #1-3, #4-6, #7-9, #11-13, #14-16 and #17-19 are correlated. The correlation structures are estimated by two factor covariance from the sample covariances of the gene sets with *p* = 30 and 120 in the diabetes dataset. We marked correlated gene sets in Table 1.

• *Setting 3*: This setting is identical to Setting 2 except that we added additional 4500 null genes to the 500 genes in the 20 gene sets. This setting mimics more practical gene expression studies. This gives a total of 5000 genes, with 104 signal genes spreading across 8 gene sets and 4896 null genes. Among the 20 gene sets, same as before, there are 8 gene sets under the alternative and 12 null gene sets.

**Table 1:** The simulation results comparing size and power of TEGS and GSEA

	**Alternative, *p * = 10**				**Null, *p * = 10**						**Alternative, *p * = 40**				**Null, *p * =40**						
		**Correlated**			**Correlated**			**Correlated**				**Correlated**			**Correlated**			**Correlated**			
No. of *β* = -1.0		0	0	1	2	0	0	0	0	0	0	0	0	2	5	0	0	0	0	0	0
No. of *β* = 1.0		0	0	3	6	0	0	0	0	0	0	0	0	8	25	0	0	0	0	0	0
No. of *β* = -0.5		1	2	0	0	0	0	0	0	0	0	2	5	0	0	0	0	0	0	0	0
No. of *β* = 0.5		3	6	0	0	0	0	0	0	0	0	8	25	0	0	0	0	0	0	0	0
No. of *β* = 0		6	2	6	2	10	10	10	10	10	10	30	10	30	10	40	40	40	40	40	40
TEGS(True)	Permutation	21.2	32.8	70.3	87.7	4.8	5.3	5.5	5.3	5.3	5.3	27.6	65.8	96.4	98.4	5.4	4.1	4.9	5.6	4.5	5.7
	$\kappa \chi_{\nu }^{2}$	22.0	33.9	70.9	88.0	4.8	5.7	5.5	5.9	5.3	5.6	27.7	67.8	96.6	99.4	5.9	4.2	4.9	5.9	4.8	5.6
TEGS(Indpt)	Permutation	11.0	20.0	41.5	75.0	6.0	6.4	5.1	6.4	5.8	5.5	9.3	23.5	34.8	91.7	3.9	6.6	4.9	3.4	6.1	5.6
	$\kappa \chi_{\nu }^{2}$	10.4	19.1	41.1	75.9	5.3	6.2	4.9	5.5	6.2	4.8	10.3	24.5	35.7	93.0	5.1	6.9	5.4	4.0	6.3	5.8
TEGS(Unstr)	Permutation	15.2	21.9	46.4	73.4	4.8	6.5	5.0	4.7	6.2	5.0	16.8	41.8	68.3	98.9	4.6	5.9	3.7	4.9	5.7	6.2
	$\kappa \chi_{\nu }^{2}$	13.0	20.8	45.1	73.0	4.3	6.1	4.6	4.4	5.7	4.9	17.5	42.4	70.5	98.9	5.5	6.1	5.0	5.2	6.4	6.4
TEGS(F-2)	Permutation	15.3	25.4	51.2	79.0	5.7	6.0	5.6	5.2	5.1	5.1	17.9	49.3	79.1	99.7	4.3	5.2	5.1	4.6	4.7	5.6
	$\kappa \chi_{\nu }^{2}$	15.4	24.5	50.5	78.4	5.6	5.6	5.3	4.8	5.0	5.2	19.9	50.7	80.8	99.8	4.7	5.2	5.0	4.9	6.0	6.2
TEGS(F-adpt)	Permutation	14.5	24.1	48.3	75.9	4.6	7.0	5.1	5.3	6.4	5.3	18.2	45.8	73.7	99.5	4.9	5.9	4.3	5.2	5.5	6.3
	$\kappa \chi_{\nu }^{2}$	13.7	22.8	48.0	75.1	5.0	6.5	4.5	5.1	5.9	5.0	18.9	46.1	74.7	99.6	4.9	5.5	5.4	5.6	5.2	6.5
TEGS(CpSym)	Permutation	14.5	24.6	53.1	84.1	5.2	5.6	5.6	5.3	5.8	5.9	16.5	34.5	71.1	99.1	4.2	4.5	4.4	3.1	5.0	5.1
	$\kappa \chi_{\nu }^{2}$	15.2	24.2	52.3	83.1	4.9	5.3	5.0	5.4	5.0	5.1	16.3	36.7	72.1	99.2	4.3	4.6	4.8	3.9	5.3	5.1
GSEA, no. of genes = 500		2.9	5.6	6.2	17.2	3.9	3.2	3.1	4.2	3.1	3.4	3.2	15.3	6.1	81.4	4.2	4.0	4.3	3.7	3.9	3.4
GSEA, no. of genes = 5,000		6.4	7.7	8.1	18.5	5.3	5.4	5.6	4.8	4.7	5.4	5.1	13.1	6.1	39.4	4.4	4.8	4.2	4.6	4.9	4.4

The entries are the percentage of times the p-values are less than 0.05. The columns with non-zero *β*’s represent the setting under the alternative and those with zero *β*’s represent the settings under the null. Abbreviations of the working covariance are the same as those in Figure 1. Two settings are considered: the first has 500 genes with 20 gene sets (8 gene sets under the alternative and 12 null gene sets of sizes 10 and 40); the second setting has 5000 genes with 20 gene sets (8 gene sets under the alternative and 12 null gene sets of sizes 10 and 40) and 4500 additional null genes. The power of TEGS for a given gene set is the same for the two settings.

For each setting, we applied TEGS and GSEA to each of the 20 gene sets to compare size and power.

### Application: reanalysis of Type II Diabetes data

We applied the proposed method to analyze the Type II Diabetes gene expression data, which were previously analyzed by Mootha *et al.* (2003) using GSEA to study for the pathway effects. The original data have three patient groups: normal glucose tolerance, impaired glucose tolerance, and Type II Diabetes. To illustrate our method and compare it with GSEA, we restricted our analysis to two groups: 17 patients with normal glucose tolerance and 17 patients with Type II Diabetes. A total of 124 out of 149 gene sets were analyzed here. We excluded 25 small gene sets, which have less than four probes.

We performed TEGS assuming five different working covariances, including independence, unstructured covariance, factor analysis covariance using two factors and the number of factors that explain up to 80% variability, and compound symmetry covariance. We calculated the p-values using permutation and the Satterthwaite method described in Section Null distribution of TEGS. The number of permutations for each gene set was 2000. The working independence TEGS corresponds to the global test [4]. We compared the performance of TEGS with GSEA. The q value, an index measuring the false discovery rate (FDR) [21,22], was used to adjust for multiple comparisons.

## Results

### Simulation study

#### Single gene set

Four true covariances were considered in the simulations: compound symmetry, AR1, two factor, and unstructured covariance. The results are presented in Figures 1, 2, 3 and 4. For each true covariance, we compared the powers of TEGS assuming the true covariance and five different working covariances.

Type I error rate is well protected at the size of 5% in all the settings with different approximation methods. For lower levels of the size (0.5% and 0.05%), different approximation methods perform well when using the true covariance (see Additional file 1: Table S1). For different working correlations, the permutation method protects the type I error at 0.5% and 0.05% where the type I error rate using the Satterthwaite approximation is inflated at 0.5% and 0.05% and that using the normal mixture approximation is well-protected at 0.5% but inflated at 0.05%.

In all settings, TEGS assuming the true covariance (the black solid line) has the best power, while TEGS assuming independence among the genes (the black dash line), has the lowest power. TEGS calculated by accounting for within-gene set correlation using an estimated working covariance is less powerful than that assuming the true covariance, but more powerful than TEGS assuming independence among the genes. As TEGS assuming independence among the genes is the same as the global test the results indicate that TEGS accounting for correlation among genes in a gene set is more powerful than the global test. A comparison of the top panel and the bottom panel in Figures 1 and 2 shows that the power gain of TEGS accounting for correlation among genes over the global test is more pronounced when the correlation is stronger.

When the working covariance structure is correctly specified, TEGS using the estimated covariance has the power closest to that using the true covariance. For example, when the true covariance is compound symmetry, TEGS assuming the compound symmetry structure with the constant pair-wise covariance estimated from the data has the power curve closest to that assuming the true compound symmetry covariance with pair-wise covariance equal to 0.1 or 0.5.

When the true covariance is the sample covariance of MAP00240_Pyrimidine_metabolism estimated from the diabetes data (Figure 4), TEGS obtained by estimating the covariance matrix using any of the working covariance matrices gives similar results, all outperforming TEGS assuming working independence among the genes (i.e., the global test). In all settings, TEGS assuming two factor analysis (F-2) and adaptive factor analysis (F-adpt) have most robust performance, and give powers that are closest to TEGS assuming the true covariance structure. The simulation results for the setting with *n* = 50 and *p* = 10 show similar patterns to those with *n* = 20 and *p* = 40, and are provided in the Additional file 1.

#### Multiple gene sets

Table 1 compares the performance of TEGS and GSEA in settings 2 and 3. The results in Table 1 show that the number of signal genes not in the gene set affects the type I error rate and power of GSEA, but does not affect TEGS. For example, when the total number of genes is 500, the size of GSEA for testing a null gene set is somewhat conservative, and less than the nominal size 0.05. As the total number of genes increased to 5000 with much more null genes, the size of GSEA for testing a null gene set became closer to 0.05.

A comparison of the powers of the 8 gene sets under the alternative show that TEGS has better power than GSEA. When the number of genes increases from 500 to 5000 with the number of signal genes remaining the same, i.e., increasing the number of null genes, the power of GSEA for testing the effect of a gene set does not improve much. The reason can be explained as below. GSEA tests for competitive null hypothesis. For a given gene set, say gene set 1, when the 4500 null genes are added to the set of 500 genes, the proportion of signal genes in gene set 1 remains the same (4/10 = 40%), while the proportion of signal genes not in the gene set decreases from 100/490 = 20% to 100/4990 = 2%. Although the difference of the proportions of signal genes in gene set 1 and not in gene set 1 becomes bigger, as the size of gene set 1 remains the same as 10, the p-value using the Kolmogorov-Smirnov test does not change much. Note that as TEGS tests for a self-contained null hypothesis [10], its power remains the same as the total number of genes increases from 500 to 5000.

The power of TEGS increases quickly with the average effect size of a gene set, $\overset{p}{\underset{j=1}{\sum }}\beta_{j}/\sigma^{2}$, while the power using GSEA improves slightly. This is because GSEA assesses whether the genes in a gene set are enriched towards the top in a list of all the genes, where individual genes are ranked by their p-values. Hence the difference between the proportion of signal genes in a given gene set and the proportion of signal genes not in the gene set affect the p-values calculated using GSEA, while the magnitudes of the signal genes have limited impact.

A comparison of the results of gene sets of size 10 with those of size 40 in Table 1 shows that the size of a gene set, *p*, affects TEGS and GSEA differently. A smaller gene set, e.g., *p* = 10, is less likely to be identified as significant using GSEA. However, the effect of the size of a gene set on TEGS is smaller. We report in Additional file 1: Table S2. The results when gene sets are independent.

To run one simulation data generated in the setting 2 (500 genes) on a desktop with 2.53 GHz CPU, the computation time of TEGS and GSEA (both with 200 permutations) is about 5.6 and 4 seconds, respectively. For the setting 3 (5000 genes), the computation time of TEGS and GSEA is about 60 and 14 seconds, respectively. The most computation burden in TEGS is to invert the working covariance in each permutation ($V_{n}^{-1}$ in (5)). Thus, the analysis with larger gene sets can cost much more computation.

### Application: re-analysis of Type II Diabetes data

TEGS assuming independence among the genes identified 5 and 8 gene sets with p values less than 0.05 using permutation and Satterthwaite methods, respectively. The corresponding numbers of gene sets were 13 and 14; 15 and 14; and 9 and 10 using TEGS by estimating the covariance assuming the two factor analysis covariance, the adaptive factor analysis covariance, and the unstructured sample covariance. GSEA identified 4 differentially expressed gene sets. The over-lapping numbers of differentially expressed gene sets between TEGS using the four working covariances and GSEA are shown in Table 2. TEGS assuming adaptive factor analysis covariance identified 10 gene sets with FDR less than 0.1 and 20 gene sets with FDR less than 0.15.

**Table 2:** Results of re-analyses of 124 gene sets in Type II Diabetes data

	**Indpt**	**Unstr**	**F-2**	**F-adpt**	**CpSym**	**GSEA**
**Permutation**						
Indpt	5	1	2	2	5	1
Unstr		9	5	7	1	0
F-2			13	9	2	1
F-adpt				15	2	1
CpSym					9	1
GSEA						4
**Scaled** ***χ***^***2***^**approximation**						
Indpt	8	1	2	2	7	2
Unstr		10	5	8	1	0
F-2			14	8	2	1
F-adpt				14	2	1
CpSym					7	1
GSEA						4

Each cell indicates the over-lapping number of differentially expressed gene sets at the nominal p-value=0.05 level using TEGS with different working covariance and GSEA shown in the corresponding column and row.

**Figure 5:**
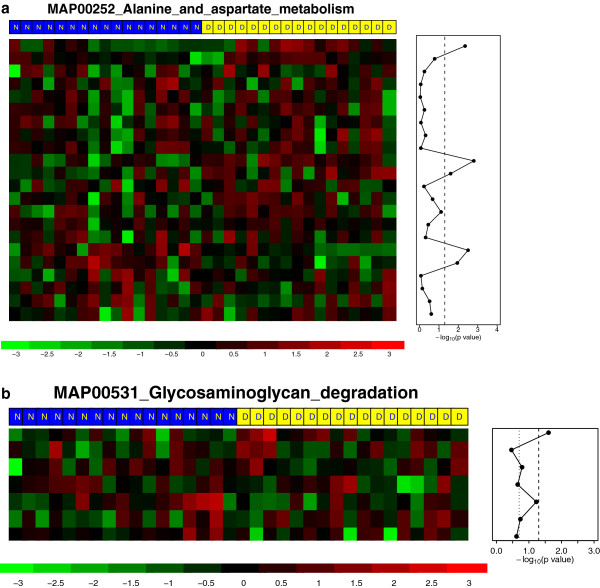
**Gene expression values in two gene sets, (a) MAP00252_Alanine_and_aspartate_metabolism and (b) MAP00531_Glycosaminoglycan_degradation.** The main plot is the expression values of genes standardized by the means and standard deviations. Each column represents a patient labeled by either “N” (normal glucose tolerance) or “D” (Type II Diabetes). The right panel represents the p values from t-test of single gene analysis where the dashed line indicates p = 0.05 and the dotted line indicates p = 0.2.

The gene set MAP00252_Alanine_and_aspartate_metabolism was identified as differentially expressed between Type II Diabetes patients and those with normal glucose tolerance: p-value=0.006 using TEGS assuming adaptive-factor covariance, p = 0.003 using TEGS with exchangeable covariance, p-value = 0.005 with TEGS with independence covariance and 0.054 using TEGS with unstructured covariance; p = 0.028 using GSEA. Figure 5a shows that five genes in this gene set were differentially expressed based on single-gene analysis with the *t*-test. The heatmap in Figure 5a also show that diabetes patients have higher expression in the upper two third of the genes but lower expression in the lower one third. Another interesting gene set we identified is MAP00531_Glycosaminoglycan_degradation (Figure 5b), which was found statistically significant using TEGS with different working correlations: p-values are 0.00034 (adaptive-factor covariance), 0.0032 (unstructured), 0.021 (independence) and 0.022 (exchangeable), but was not significant using GSEA: p-value=0.39.

## Discussion

The power of TEGS is improved by accounting for the correlation among genes within the gene set, especially when the working covariance gets closer to the true covariance, and outperforms the TEGS assuming working independence. We have also shown that the TEGS with working independence among genes in a gene set corresponds to the global test proposed by Goeman, *et al* (2004). Our numerical studies show that the TEGS assuming two factors or adaptive factor covariance matrix overall works well in practice for difference true covariance structures, especially when the number of genes *p* is larger than sample size *n*.

We have compared the performance of TEGS with GSEA. Both tests borrow information across multiple genes in a gene set and are hence beneficial when a gene set has multiple signal genes with modest effects. TEGS and GSEA differ in several aspects. The TEGS statistic is constructed by accounting for correlation among genes in a gene set, while GSEA uses individual gene p-values to calculate the Kolmogorov-Smirnov test, which ignores the within gene set correlation. TEGS considers the *self-contained* null hypothesis, while GSEA considers the *competitive* null hypothesis [10]. GSEA studies the enrichment of genes in a gene set by testing the relative rankings of the genes in a gene set among all the genes under investigation. GSEA hence is influenced by the size of a gene set, the proportion of signal genes in the gene set, and the proportion of signal genes not in the gene set. When the proportion of signal genes in a gene set is much larger relative to that not in the gene set, GSEA performs well. We note that GSEA has difficulties in capturing a differentially expressed gene set when the number of genes containing true effects is small in a gene set even if the effects of these signal genes are strong. When the size of a gene set is small/modest, the power of GSEA does not increase much when the number of null genes not in the gene set increases or when the sample size increases. As TEGS considers a self-contained null hypothesis, i.e., testing whether a gene set is differentially expressed, it is not affected by the behavior of the genes not in the gene set. TEGS improves power when sample size increases or the magnitudes of signal genes in a gene set increase. However, TEGS does not directly compare a gene set with other gene sets, although one can rank gene sets using p-values calculated using TEGS. Our numerical results show that TEGS outperforms GSEA in terms of size and power, although the powers are not directly comparable as they test for different null hypotheses.

Due to the nature of the null hypothesis we specified, it is possible that a significant gene set from our proposed testing procedure is driven by one or two very significant genes, which is less likely to occur in GSEA. There are several possible ways to guard against this. For example, after a gene set is identified to be significant, one can perform single gene analyses to further characterize how the signals are distributed within the gene set. Or, one can use the same multivariate model as (1) to estimate and test the association of each gene with the phenotype using the ridge regression.

TEGS is not limited to testing mRNA expression in biological pathways/networks. It can also be applied for testing the effects of other genomic markers, such as DNA copy numbers, RNA or protein expressions, and DNA methylations in a genomic region or a functional set.

## Conclusions

We have proposed in this paper a new method for the gene set analysis, TEGS. By introducing a working covariance, TEGS directly models the interdependence of the expression values in a gene set, the most important feature of biological pathways or gene sets that is often overlooked in existing methods. TEGS incorporates information from multiple genes in a gene set through the working covariance and thus outperforms two widely used approach, GSEA and global test in simulation studies and a diabetes microarray data.

## Competing interests

Both authors declare that they have no competing interests.

## Authors’ contributions

YTH developed the statistical methodology, designed and implemented the methods in analyzing microarray data and simulation data, and wrote the manuscript. XL developed the methodology and helped to draft and revise the masnucript. Both authors read and approved the final manuscript.

## Supplementary Material

Additional file 1 Supplementary results. Click here for file
