# Supplementary material for: Gene set analysis using variance component tests
Source: BMC Bioinformatics. 2013 Jun 28;14:210. doi: 10.1186/1471-2105-14-210 (PMC3776447; doi:10.1186/1471-2105-14-210)
Supplement: Additional file 1 — Supplementary results. [file 1471-2105-14-210-S1.pdf]

Supplementary Material to 'Gene set analysis using variance component tests'

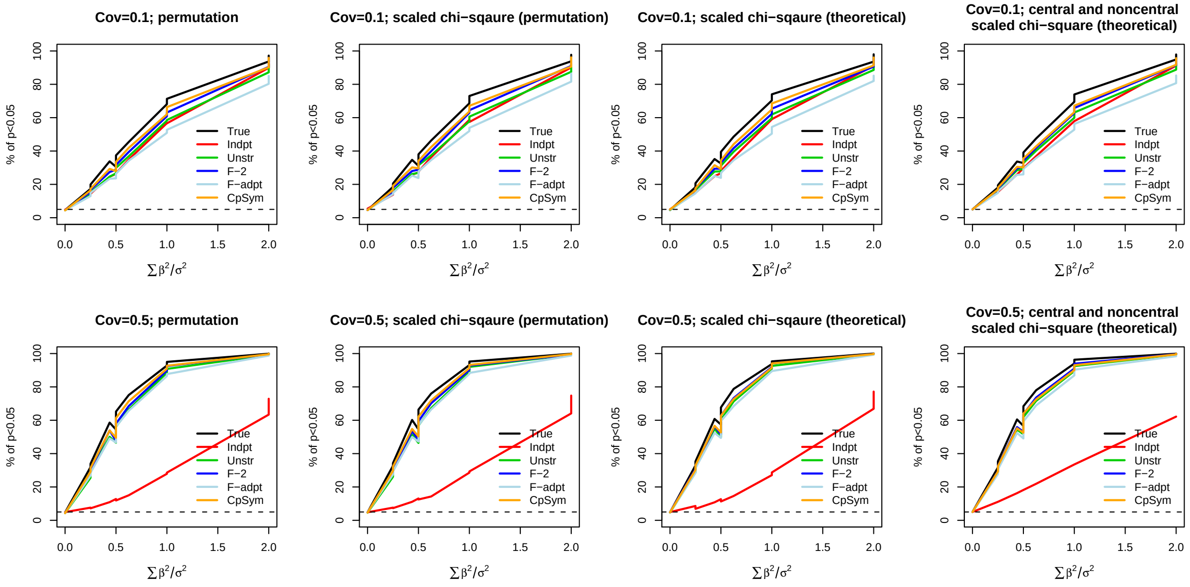

Figure S1: The power plots from the simulations by the CS true covariance and n=50 & p=10.

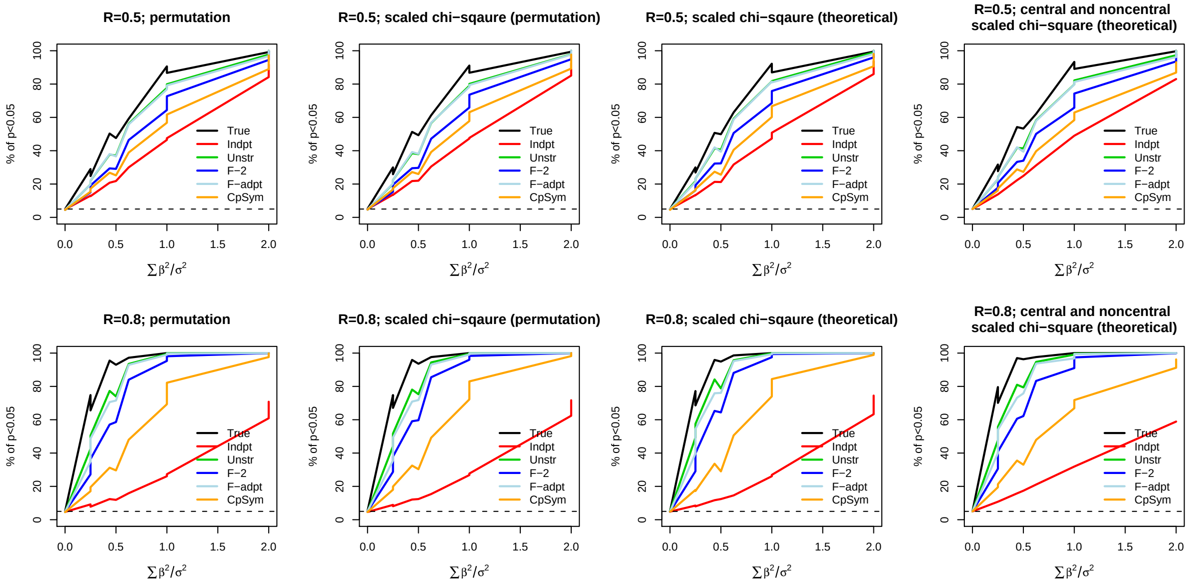

Figure S2: The power plots from the simulations by the AR1 true covariance and n=50 & p=10.

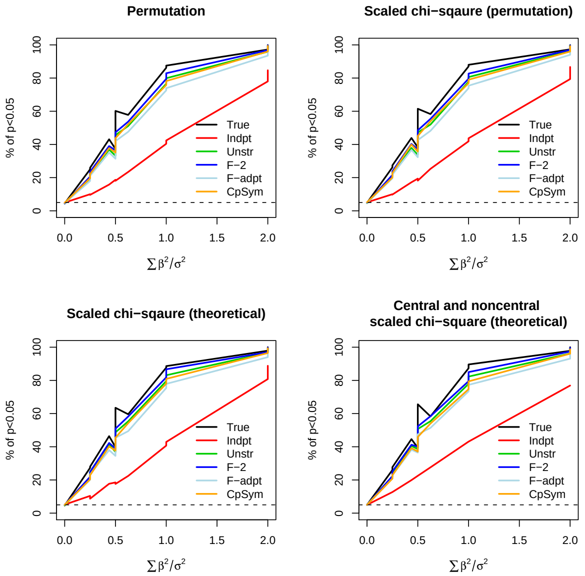

Figure S3: The power plots from the simulations by the F2 true covariance and n=50 & p=10.

Table S1: Empirical sizes (%) of TEGS with different working covariances (true covariance [True], independence [Indpt], unstructured covariance [Unstr], two-factor covariance [F-2] and compound symmetry covariance [CpSym]) using different approximation methods (permutation, scaled χ^2 approximation and normal mixture approximation. The empirical size was calculated at the significance level of 5%, 0.5% and 0.05% based on 10,000 simulations. The scaled χ^2 and normal mixture approximation were based on 1000 permutations.

Significance level	True	Indpt	Unstr	F-2	CpSym
Permutation					
5%	4.97	5.05	4.87	4.97	4.96
0.5%	0.53	0.43	0.55	0.51	0.42
0.05%	0.07	0.05	0.08	0.04	0.05
Scaled χ^2 approximation					
5%	5.05	4.99	5.50	5.73	5.07
0.5%	0.49	0.91	1.00	1.09	0.96
0.05%	0.04	0.28	0.23	0.29	0.25
Normal mixture approximation					
5%	5.11	4.60	5.28	5.36	5.06
0.5%	0.63	0.52	0.56	0.65	0.54
0.05%	0.09	0.13	0.14	0.19	0.08

Table S2 : The result of simulation studies of uncorrelated multiple gene sets (Setting 1 in the main text). The gray cell represents that the gene set is under the alternative with the indicated β's where the white cell represents the null. Abbreviations are the same as indicated in Figure 1 of the main text.

n=20		Alternative, p=10				Null, p=10						Alternative, p=40				Null, p=40					
		uncorrelated																			
% of p<0.05	No. of β=-1.0	0	0	1	2	0	0	0	0	0	0	0	0	2	5	0	0	0	0	0	0
	No. of β=1.0	0	0	3	6	0	0	0	0	0	0	0	0	8	25	0	0	0	0	0	0
	No. of β=-0.5	1	2	0	0	0	0	0	0	0	0	2	5	0	0	0	0	0	0	0	0
	No. of β=0.5	3	6	0	0	0	0	0	0	0	0	8	25	0	0	0	0	0	0	0	0
	No. of β=0	6	2	6	2	10	10	10	10	10	10	30	10	30	10	40	40	40	40	40	40
True	Permutation	20.7	26.7	70.4	99.5	5.6	5.7	5.8	5.4	5.4	5.3	29.6	53.2	94.4	100.0	4.8	4.3	4.8	5.2	5.4	5.2
	$\nu\chi^2$ (permutation)	21.3	27.5	71.7	99.5	5.7	6.0	5.4	5.6	5.8	5.6	30.1	55.7	94.9	100.0	4.8	4.5	5.1	5.7	5.4	5.3
	$\nu\chi^2$ (theoretical)	20.5	28.7	76.6	99.7	4.7	4.8	4.8	4.1	4.3	4.7	32.2	56.0	95.4	100.0	3.4	4.3	4.8	4.1	5.1	4.7
	$\nu\chi^2(\delta)$ (theoretical)	21.5	29.6	77.1	99.9	5.0	5.0	5.0	5.0	5.0	5.0	30.6	60.1	96.2	100.0	5.0	5.0	5.0	5.0	5.0	5.0
Indpt	Permutation	10.8	21.3	41.2	82.0	5.7	5.4	5.0	5.3	5.4	5.6	10.4	26.6	38.4	90.9	5.5	4.5	5.7	5.6	5.9	4.1
	$\nu\chi^2$ (permutation)	11.3	21.2	41.5	82.1	5.4	5.4	4.4	4.8	5.4	5.2	11.0	27.5	39.2	92.3	6.3	5.1	6.0	6.6	5.4	4.5
	$\nu\chi^2$ (theoretical)	11.2	22.3	47.7	87.8	4.7	4.6	4.4	4.9	4.9	4.8	12.2	29.8	41.7	95.9	5.6	5.5	6.0	6.1	4.3	5.5
	$\nu\chi^2(\delta)$ (theoretical)	13.6	25.3	49.0	81.5	5.0	5.0	5.0	5.0	5.0	5.0	13.3	33.9	44.0	92.5	5.0	5.0	5.0	5.0	5.0	5.0
Unstr	Permutation	15.1	22.2	50.4	91.6	4.7	5.2	4.8	5.3	5.0	5.1	18.2	41.8	71.8	99.2	4.8	5.8	5.3	5.1	5.4	5.2
	$\nu\chi^2$ (permutation)	14.7	21.7	48.7	90.4	5.0	5.1	4.6	5.1	4.5	5.1	19.2	42.4	73.3	99.3	5.0	6.5	5.6	4.9	5.7	5.8
	$\nu\chi^2$ (theoretical)	17.7	26.4	58.6	94.5	5.3	5.3	5.4	5.2	5.0	5.0	22.0	48.4	83.5	99.7	4.9	4.2	5.8	5.2	4.4	5.0
	$\nu\chi^2(\delta)$ (theoretical)	18.1	26.1	58.9	92.2	5.0	5.0	5.0	5.0	5.0	5.0	20.9	50.8	82.6	99.8	5.0	5.0	5.0	5.0	5.0	5.0
F−2	Permutation	16.0	23.4	54.1	95.0	6.1	4.8	5.3	5.3	5.4	5.5	20.3	47.4	83.9	99.9	4.2	4.8	5.2	5.7	5.0	4.4
	$\nu\chi^2$ (permutation)	15.4	23.4	53.9	94.7	5.8	4.9	4.9	5.3	5.6	5.3	20.6	48.4	84.1	99.9	5.1	5.0	5.5	5.3	5.7	4.8
	$\nu\chi^2$ (theoretical)	17.1	28.4	62.8	97.4	5.4	5.0	5.3	5.0	4.7	4.8	24.6	56.0	91.4	100.0	4.9	5.5	5.5	5.5	5.0	6.0
	$\nu\chi^2(\delta)$ (theoretical)	19.2	28.6	64.3	96.1	5.0	5.0	5.0	5.0	5.0	5.0	23.5	57.8	90.2	100.0	5.0	5.0	5.0	5.0	5.0	5.0
F−adpt	Permutation	16.0	22.4	52.9	91.5	6.0	5.8	5.3	5.9	5.6	5.6	18.8	44.4	78.2	99.6	5.6	6.1	5.6	5.6	5.3	4.8
	$\nu\chi^2$ (permutation)	15.1	22.0	52.6	91.5	5.8	5.7	5.1	5.6	5.2	5.4	20.0	45.7	79.4	99.6	5.9	6.6	6.3	6.1	5.7	5.6
	$\nu\chi^2$ (theoretical)	17.4	25.6	59.5	93.0	5.0	5.6	5.9	4.9	5.1	4.6	23.1	54.8	88.9	100.0	4.8	4.9	5.2	5.2	4.7	6.0
	$\nu\chi^2(\delta)$ (theoretical)	18.5	26.4	60.1	92.6	5.0	5.0	5.0	5.0	5.0	5.0	22.6	55.7	87.5	99.9	5.0	5.0	5.0	5.0	5.0	5.0
CpSym	Permutation	16.7	27.3	56.5	89.8	5.9	6.1	5.9	5.9	5.6	5.7	16.5	41.7	76.2	99.6	4.6	5.1	6.3	4.8	4.3	4.4
	$\nu\chi^2$ (permutation)	16.4	26.5	55.3	89.7	5.3	5.5	5.3	5.5	5.3	5.5	17.5	42.5	77.2	99.3	4.5	5.3	6.7	5.0	4.4	4.6
	$\nu\chi^2$ (theoretical)	17.8	29.4	62.4	92.6	4.3	4.2	4.6	4.8	4.5	4.9	19.2	45.2	79.6	99.7	5.1	5.2	6.1	4.8	5.0	4.4
	$\nu\chi^2(\delta)$ (theoretical)	19.0	31.1	63.1	89.3	5.0	5.0	5.0	5.0	5.0	5.0	19.7	47.9	78.6	99.3	5.0	5.0	5.0	5.0	5.0	5.0
GSEA		7.2	5.2	5.0	5.2	5.2	4.9	5.1	4.9	5.2	5.0	4.8	4.6	4.9	5.6	4.5	4.4	4.8	4.7	4.6	4.3
		4.8	14.8	5.0	5.1	5.2	5.0	5.1	4.8	5.2	5.0	4.6	4.6	4.7	5.6	4.5	4.3	4.6	4.4	4.5	4.0
		4.8	5.0	12.9	5.1	5.0	4.9	5.0	4.9	5.2	5.0	4.5	4.4	4.6	5.5	4.5	4.3	4.7	4.4	4.4	4.0
		4.8	5.0	5.0	32.5	5.0	4.9	4.9	4.8	5.1	4.9	4.1	4.3	4.3	4.9	4.2	3.9	4.4	4.0	4.2	3.8
		4.8	5.0	4.8	5.0	5.0	4.7	4.9	4.8	5.1	4.7	14.3	4.5	4.6	5.4	4.4	4.4	4.8	4.3	4.5	4.2
		4.4	4.7	4.5	4.7	4.5	4.5	4.7	4.5	4.9	4.3	4.5	50.1	4.4	5.3	4.2	4.4	4.7	4.3	4.2	4.1
		4.5	4.7	4.6	4.7	4.6	4.5	4.8	4.6	4.9	4.4	4.4	4.2	29.0	5.3	4.2	4.3	4.7	4.2	4.3	4.0
		3.8	4.0	4.0	3.9	3.9	3.9	3.8	4.0	4.3	3.8	4.2	3.9	4.1	83.4	3.8	4.1	4.3	3.9	3.8	3.8
		6.7	13.2	11.5	29.5	5.0	4.9	5.1	4.8	5.1	4.8	3.7	3.9	3.8	4.5	3.7	3.4	4.0	3.6	3.8	3.3
		3.4	3.7	3.6	3.7	3.5	3.6	3.6	3.8	3.8	3.5	4.5	40.6	4.5	83.1	4.1	4.5	4.7	4.0	4.0	4.2
		3.2	6.4	5.5	19.2	3.2	3.2	3.5	3.6	3.4	3.2	3.1	29.1	11.0	78.4	4.1	4.6	4.6	3.7	3.8	4.1
